# The Effect of Exercise Intensity on Gastric Emptying Rate, Appetite and Gut Derived Hormone Responses after Consuming a Standardised Semi-Solid Meal in Healthy Males

**DOI:** 10.3390/nu10060787

**Published:** 2018-06-19

**Authors:** Lewis R. Mattin, Adora M. W. Yau, Victoria McIver, Lewis J. James, Gethin H. Evans

**Affiliations:** 1 School of Healthcare Science, Manchester Metropolitan University, Manchester M1 5GD, UK; L.Mattin@mmu.ac.uk (L.R.M.); A.Yau@mmu.ac.uk (A.M.W.Y.); victoria.mciver@stu.mmu.ac.uk (V.M.); 2 School of Sport, Exercise and Health Sciences, Loughborough University, Loughborough LE11 3TU, UK; L.James@lboro.ac.uk

**Keywords:** aerobic exercise, cycling, appetite, gastric emptying, gut hormones, ghrelin

## Abstract

This study investigated the acute circulating gut hormone, appetite and gastric emptying rate responses to a semi-solid meal following exercise at different intensities. Twelve men completed three trials in a randomised-crossover design, consisting of continuous cycling at 70% $\dot{V}$O_2Peak_ (HIGH), 40% $\dot{V}$O_2Peak_ (LOW) or rest (CONTROL). Baseline samples were collected after an overnight fast before undertaking the 60 min exercise or rest period, followed by 30 min rest before consumption of a standardised semi-solid meal (~242 kcal). During the 2 h postprandial period, gastric emptying rate of the meal was examined using the ^13^C-breath test method, appetite was measured using visual analogue scales, and serum concentrations of acylated ghrelin, pancreatic polypeptide, peptide YY, glucagon-like peptide-1, insulin, glucose, triglycerides, total cholesterol and non-esterified fatty acids were assessed. Subjective appetite response was not different between trials (*p* > 0.05). Half emptying time of the meal was 89 ± 13, 82 ± 8 and 94 ± 31 min on CONTROL, LOW and HIGH, respectively (*p* = 0.247). In healthy un-trained adult males, responses to exercise at intensities of 70% and 40% $\dot{V}$O_2Peak_ did not differ to a non-exercise control for measurements of subsequent gastric emptying, circulating gut hormone response or appetite. These results suggest that exercise intensity has little effect on post-exercise appetite response to a semi-solid meal.

## 1. Introduction

As the prevalence of obesity increases, ultimately caused by a chronic energy excess, investigations have focused on reducing energy intake and/or increasing exercise energy expenditure as an effective weight loss strategy. Understanding how exercise influences subjective feelings of appetite is important so that subsequent energy intake does not exceed the energy expended by physical activity and potentially lead to positive energy balance [1].

It has been suggested that exercise may stimulate compensatory increases in appetite and food intake that attenuates weight loss; however, this evidence is limited [2,3]. Previous investigations have demonstrated that high-intensity [4,5,6,7,8], but not low intensity [1,9,10,11] aerobic exercise suppresses appetite immediately after exercise. Ueda et al. [12] observed that 60 min cycling at 50% maximum oxygen uptake ($\dot{V}$O_2max_) did not stimulate appetite. Conversely, strenuous exercise >60% of $\dot{V}$O_2max_ has been consistently shown to suppress appetite for up to 30 min after an exercise bout [13,14,15]. This effect on subjective feelings of appetite is unlikely to influence longer term energy intake, regardless of the increased metabolic response of the exercise compared to a lower intensity exercise <40%$\;\dot{V}$O_2max_, but may influence appetite regulation following the ingestion of food in the period after exercise. A number of studies have shown that exercise results in little change in post exercise energy intake (EI), with the typical method used to assess energy intake involving *ad libitum* meals [1,5,16,17]. However, providing an *ad libitum* meal after exercise could be seen as unrealistic as energy intake within these ingestion periods can be as high as ~5500 kJ [7].

Energy homeostasis is regulated both centrally and peripherally [18]. Within this complex system peptides are secreted from the gastrointestinal tract with literature focusing on ghrelin, peptide tyrosine tyrosine (PYY), glucagon-like peptide-1 (GLP-1), and pancreatic polypeptide (PP) in response to nutrient ingestion after moderate intensity exercise [13,19]. However, most studies tend to investigate the effect of exercise on one or two of these hormones when many have been shown to influence appetite [4,8,10,20]. However, Martins et al. [13] measured multiple gastrointestinal hormones, and observed that cycling at a moderate intensity for 60 min increased PYY, GLP-1 and PP and decreased ghrelin. Of these, gut peptides ghrelin still remains unique as the only known appetite stimulating gut peptide. In addition, gastric emptying has been suggested as a rate-limiting step in the delivery of nutrients to the small intestine and an important understudied factor in appetite control [21,22]. Evans et al. [23] investigated the effect of exercise intensity on gastric emptying rate post exercise, and found that gastric emptying rate was unaffected after consuming a 5% glucose solution. It is unknown whether exercise intensity affects the gastric emptying rate of a semi-solid meal. Therefore, the aim of this investigation was to examine the effect of exercise intensity on gastric emptying rate, appetite and gut-derived hormone response to a standardised semi-solid meal.

## 2. Methods

### 2.1. Participants

Twelve healthy men matched the criteria (age = 18–40 years; body mass index ≤ 29.9 kg/m^2^; non-smokers, no history of gastrointestinal symptoms or disease, not consuming medication, had no other relevant medical conditions assessed by a medical screening questionnaire and be habitually physically active). Verbal and written explanations of the experimental procedures were given before the start of any trial and written informed consent to participate was obtained. Ethical approval was provided via the Ethical Advisory Committee of Manchester Metropolitan University Faculty of Science and Engineering (Reference Number: SE151667).

### 2.2. Familiarization

Before undertaking the main trials, participants visited the laboratory to undergo familiarization, anthropometric and exercise testing. Questionnaires were completed including, health screening, physical activity and dietary habits. Height was measured to the nearest 0.1 cm using a wall-mounted stadiometer and body mass to the nearest 0.01 kg using electronic scales (GFK 150; Adam Equipment Co. Ltd., Milton Keynes, UK). Body fat percentage was approximated using bioelectrical impedance analysis (Bodystat 1500, Body Composition Technology, Isle of Man, UK). Participants were then familiarized with the gastric emptying assessment technique and visual analogue scale (VAS) to be used during the experimental trials. The VAS was composed of questions asking: “How hungry do you feel?” “How full do you feel?” “How much do you think you can eat?” and “How satisfied do you feel?” [24]. Horizontal lines 100 mm in length were anchored with “I am not hungry at all to I have never been more hungry,” “Not at all full to totally full,” “nothing at all to a lot,” and “I am completely empty to I can’t eat another bite” at 0 mm and 100 mm, respectively.

Peak oxygen uptake ($\dot{V}$O_2Peak_) was measured on a cycle ergometer (Lode Excalibur Sport, Groningen, The Netherlands). VO_2_ and VCO_2_ measurements were taken by indirect calorimetry and heart rate (HR) (Polar FS2c, Kemple, Finland) recorded. Workload began at 50 W and a cadence of 70 revolutions/min was maintained throughout. Workload was then increased in increments of 50 W, every 3 min, until participants began to show signs of fatigue (assessed through heart rate and rating of perceived exertion). At this point, the workload was increased by 20 W every minute until volitional exhaustion. $\dot{V}$O_2Peak_ was calculated from the last 1 min before exhaustion. Workload during the main trial (40% and 70% $\dot{V}$O_2Peak_) were calculated from maximum workload (W) at $\dot{V}$O_2Peak_. Before leaving the laboratory, participants were given food scales (Salter, ARC 1066 Electronic Kitchen scale Max 3 kg, Tonbridge, UK) and a physical activity and food diary.

### 2.3. Experimental Protocol

Experimental trials were conducted in a randomised-crossover design commencing between 0800 and 0900 following an overnight fast having consumed only water from 2200 the previous evening. Experimental trials were separated by a minimum of 7 days. Each experimental trial was preceded by 24 h weighed food and drink intake and an activity maintenance period during which participants were asked to record in their diet and activity diary before the first trial, and then replicate these patterns prior to the next two trials. To ensure that participants were adhering to the dietary standardisation procedures, the research team contacted participants via telephone the day before each main trial. The purpose of this was to ensure standardisation and consistency of macronutrient intake and metabolic status in the 24 h leading to each trial. Furthermore, participants were asked to refrain from alcohol, caffeine consumption and strenuous physical activity in the 24 h preceding each experimental trial. The atmospheric temperature was set at 21 °C and a relative humidity of 30% throughout the trials.

**Figure 1:**
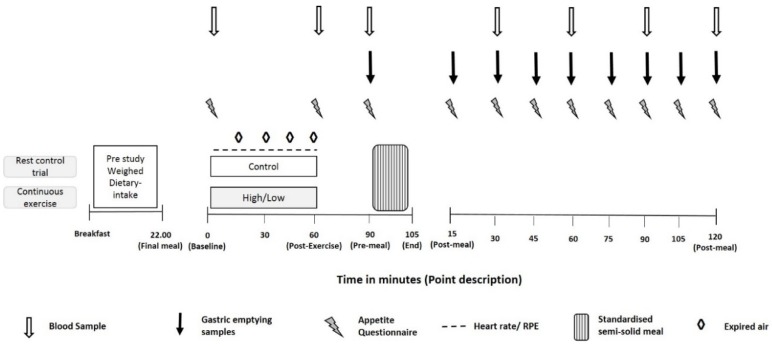
Schematic diagram of the experimental trial protocol.

Upon arrival at the laboratory, participants’ body mass was obtained after emptying their bladder and they rested for 15 min in a semi-supine position whilst a cannula was inserted into the antecubital vein to enable venous blood collection. Following collection of a blood sample, a VAS was completed. Participants then completed a 60 min period of rest (CONTROL) or cycle exercise at 40% (LOW) or 70% (HIGH) $\dot{V}$O_2Peak_. Expired air was collected and analysed for VO_2_ every 15 min during exercise or rest using Douglas bags. Heart rate and ratings of perceived exertion (Borg scale, [25]) were recorded at 15 min intervals. During the 60 min exercise trials, participants had *ad libitum* access to water. The volume of water consumed was measured by weighing the drinks bottles pre and post-exercise (Adam Equipment Co Ltd., PGL 303, Milton Keynes, UK). After completion of the exercise bout, a further blood sample was collected and VAS completed before participants were given 30 min to shower and change their clothes. Following collection of a blood sample and completion of a VAS, participants were fed a standardised meal after which they rested in the laboratory for 2 h. VAS were completed at 15 min intervals throughout the 2 h postprandial period, with blood samples collected at 30 min intervals. A schematic diagram of the experimental trial protocol is provided in Figure 1.

### 2.4. Meal

In all three trials, the same standardised meal was consumed by all participants. The meal consisted of one 400 g can of classic chicken and sweetcorn soup heated in a microwave. The meal provided 242 kcal/1006 kJ and the macronutrient content of the meal was 11.8 g fat, 8.2 g protein, 25.2 g carbohydrates, 0.8 g fibre and 2.2 g salt. The meal provided each participant with 12% of their reference energy intake of an average adult (2000 kcal). Participants had 15 min to consume the standardised meal. Time taken to eat the meal was recorded.

### 2.5. Measurement of Gastric Emptying

Gastric emptying rate was assessed using the non-invasive ^13^C-acetate breath method. Meals contained 100 mg ^13^C-sodium acetate (^1-13^C, 99%; Cambridge Isotope Laboratories Inc., Andover MA, USA), which was first dissolved in 20 mL of water before adding it to the heated soup. A basal end expiratory breath sample was collected prior to food ingestion and further breath samples were collected at 15 min intervals over the 120 min after ingestion of the test meal. Samples were analysed by non-dispersive IR spectroscopy (IRIS Dynamic, Kibion, Germany) for the ratio of ^13^CO_2_:^12^CO_2_. The difference in the ratio of ^13^CO_2_:^12^CO_2_ from baseline breath to post-ingestion breath samples are expressed as delta over baseline (DOB). Half emptying time (T_1/2_) and time of maximum emptying rate (T_lag_) were calculated using the manufacturer’s integrated software evaluation based on the equations of Goos et al. [26].

### 2.6. Blood Sampling and Analysis

All blood samples were collected with the participant in a semi-supine position and cannulas were kept patent by flushing with nonheparinized saline (0.9% sodium chloride). Upon collection of blood samples, 50 μL of Pefabloc (Roche Diagnostics Limited, Burgess Hill, UK) and 50 μL Dipeptidyl peptidase IV inhibitor (DPP-IV), (Merck Millipore Limited., Feltham UK) were immediately added to the blood to prevent the degradation of acylated ghrelin and active GLP-1. Samples were then kept on ice until they were centrifuged (Sigma 3-16KL, Osterode am Harz, Germany) at 1500 g for 15 min at 4 °C, and then immediately stored at −80 °C for later biochemical analysis. The circulating concentrations of PYY, PP, insulin, active GLP-1, and acylated ghrelin were determined using multiplex analysis (Luminex 200, Luminex Corporation, Austin, TX, USA) with human gut hormone Merck-Millipore kits (Milliplex MAP, Merck Millipore Ltd., Feltham, UK). Serum glucose, triglycerides, total cholesterol and NEFA were analysed using a clinical chemistry analyser (Randox Daytona, Crumlin, UK). Analysis was performed in duplicate.

### 2.7. Statistical Analysis

Differences in baseline measurements, T_lag_ and T_1/2_ were analysed using one-way repeated measures analysis of variance (ANOVA). Post hoc analysis consisted of Bonferroni adjusted pairwise comparisons where necessary. Two-way repeated ANOVA were used to examine differences in gastric emptying DOB values, blood hormones, metabolites and VAS scores. Sphericity for repeated measures was assessed and, where appropriate, Greenhouse–Geisser epsilons were used to correct for violations. Significant main effects were followed by paired student’s *t*-Test or one-way repeated ANOVA and Bonferroni adjusted pairwise comparisons as appropriate. All data was analysed using SPSS statistics for Windows v. 24 (IBM, New York, NY, USA). Statistical significance was accepted at the 5% level and results presented as mean ± standard deviation (SD).

## 3. Results

### 3.1. Participant Characteristics

Twelve men (mean ± SD: age, 26 ± 5 year; weight, 80.58 ± 12.74 kg; height, 1.76 ± 0.10 m; BMI, 25.5 ± 3.5 kg·m^−2^; body fat, 18.9% ± 8.1%; $\dot{V}$O_2Peak_, 42.16 ± 6.63 mL·kg^−1^·min^−1^) were recruited from central Manchester, UK. Four participants were not included in the blood parameters due to a fault with the cannula during trials.

### 3.2. Pre-Trial Standardisation, Exercise and Food Ingestion

Pre-trial energy intake for CONTROL, HIGH and LOW was not significantly different between trials (*p* = 0.968), with a similar proportion of energy from fats (35 ± 8%, 37 ± 11% and 33 ± 9%: *p* = 0.339), carbohydrates (44 ± 9%, 42 ± 9% and 46 ± 8%: *p* = 0.670) and protein (19 ± 6%, 20 ± 5% and 19 ± 6%: *p* = 0.294) before CONTROL, HIGH and LOW, respectively. In addition, fluid consumption before CONTROL, HIGH and LOW was not significantly different between trials (*p* = 0.196).

Average heart rate was significantly higher during the HIGH trial than LOW and CONTROL (*p* < 0.001) and was higher in LOW compared to CONTROL (*p* < 0.001). Water consumption during the 1 h exercise was significantly greater during the HIGH trial than the CONTROL trial (*p* = 0.026). Average temperature within the climate control room during the 1 h was not significantly different during trials (*p* = 0.182).

The weight of the soup consumed was not significantly different between trials (396 ± 2 g, 395 ± 2 g, and 397 ± 3 g for HIGH, LOW and CONTROL, respectively; *p* = 0.150). Differences in time to eat the soup between trials approached significance (HIGH 327 ± 73 s, LOW 303 ± 82 s and CONTROL 307 ± 113; *p* = 0.067).

### 3.3. Appetite and Satiety

**Figure 2:**
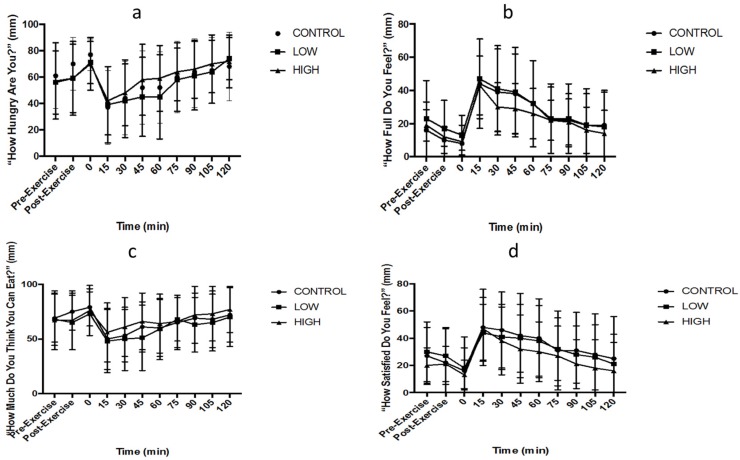
Visual analogue scale (VAS) of (**a**) hunger, (**b**) fullness, (**c**) prospective food consumption (PFC), (**d**) satisfaction. 0 min (pre-meal), and then every 15 min post-meal for 120 min. Values were mean ± standard deviation (*n* = 12).

Two-factor ANOVA demonstrated main effects of time for hunger (*p* < 0.001: Figure 2a), fullness (*p* < 0.001: Figure 2b), prospective food to be eaten (*p* = 0.008: Figure 2c) and satisfaction (*p* < 0.001: Figure 2d), but not for bloating (*p* = 0.218). Hunger and prospective food to be eaten decreased, whilst fullness and satisfaction increased after eating the meal. There were no trial or interaction effects for hunger (*p* = 0.339; *p* = 0.190), prospective food to be eaten (*p* = 0.058; *p* = 0.087), satisfaction (*p* = 0.248; *p* = 0.650) or bloating (*p* = 0.302; *p* = 0.456). There was no interaction effect for fullness (*p* = 0.456), but there was a main effect of trial (*p* = 0.025); however, post hoc tests revealed no between-trial differences.

### 3.4. Gastric Emptying

**Figure 3:**
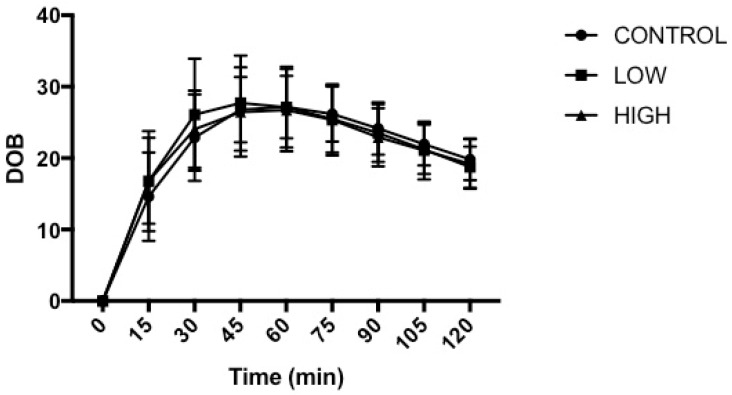
Gastric emptying rate of the standardised semi-sold meal. Delta over baseline (DOB), pre-meal (0 min), and then every 15 min post-meal for 120 min. Values were mean ± standard deviation (*n* = 12).

The time taken to empty half of the soup from the stomach (T_half_) amounted to 89 ± 13 min, 82 ± 8 min and 94 ± 31 min on CONTROL, LOW and HIGH, respectively (*p* = 0.247). The time of maximal emptying rate (T_lag_) amounted to 63 ± 13 min, 56 ± 10 min and 60 ± 16 min for CONTROL, LOW and HIGH, respectively (*p* = 0.235). Two-factor ANOVA demonstrated no main effect of trial (*p* = 0.853), a main effect of time (*p* < 0.001) and no interaction effect (*p* = 0.162) for DOB values (Figure 3). DOB values were elevated (*p* < 0.05) from pre-meal values from 15 min after soup ingestion until the end of each trial.

### 3.5. Blood Metabolites

**Figure 4:**
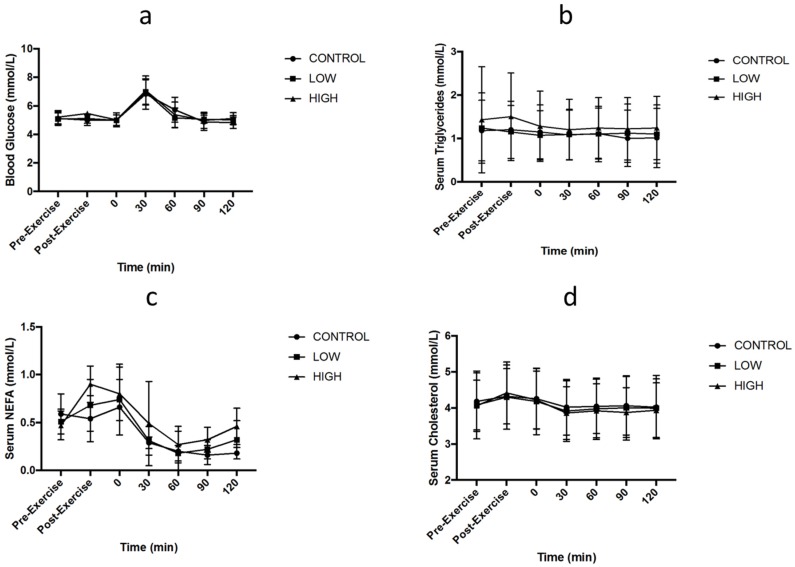
Serum concentrations of (**a**) glucose, (**b**) triglycerides, (**c**) Non-esterified fatty acids (NEFA) and (**d**) total cholesterol. 0 (pre-meal), 30 (30-min post-meal), 60 (60-min post-meal), 90 (90-min post-meal), 120 (120-min post-meal). Values are mean ± standard deviation (*n* = 8).

Two-factor ANOVA demonstrated no main effect of trial for blood glucose (*p* = 0.426: Figure 4a), circulating triglycerides (*p* = 0.197: Figure 4b), NEFA (*p* = 0.114: Figure 4c) or total cholesterol (*p* = 0.414: Figure 4d). No main effect of time was observed for circulating triglycerides (*p* = 0.107), but it was observed for blood glucose (*p* < 0.001), cholesterol (*p* < 0.001) and NEFA (*p* < 0.001). No interaction effects were observed for serum glucose (*p* = 0.215), triglycerides (*p* = 0.342), cholesterol (*p* = 0.093) or NEFA (*p* = 0.103).

### 3.6. Gut Hormones

**Figure 5:**
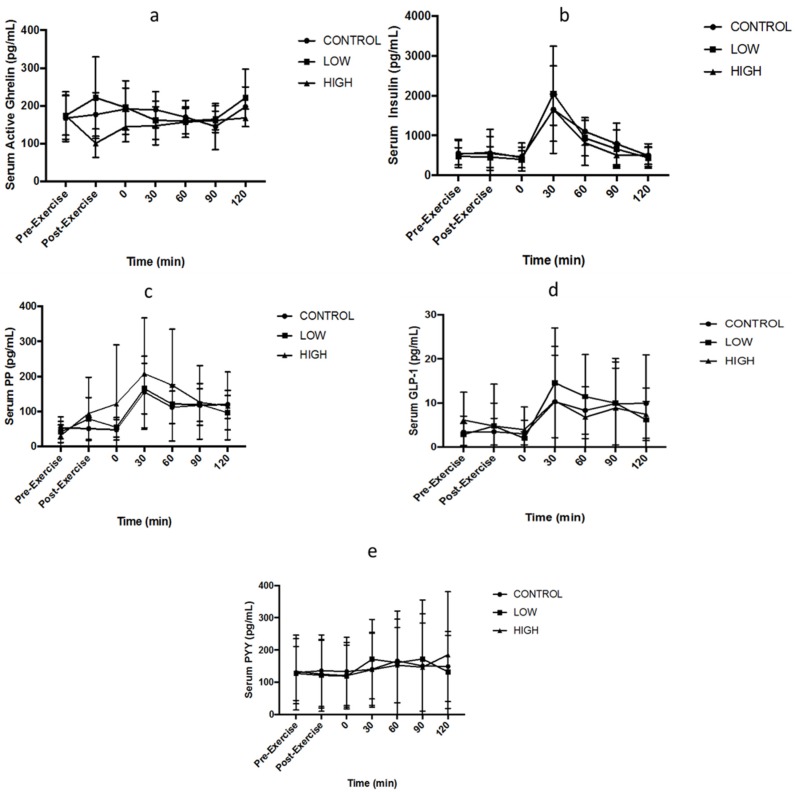
Serum concentrations of (**a**) ghrelin, (**b**) insulin, (**c**) pancreatic polypeptide (PP), (**d**) GLP-1, and (**e**) PYY. 0 (pre-meal), 30 (30-min post-meal), 60 (60-min post-meal), 90 (90-min post-meal), 120 (120-min post-meal). Values are mean ± standard deviation (*n* = 8).

Two-factor ANOVA demonstrated no main effect of trial for circulating ghrelin (*p* = 0.08: Figure 5a), GLP-1 (*p* = 0.856: Figure 5d), insulin (*p* = 0.604: Figure 5b), PP (*p* = 0.377: Figure 5c) or PYY (*p* = 0.992: Figure 5e). Main effects of time were observed for GLP-1 (*p* = 0.044), insulin (*p* < 0.001) and PP (*p* < 0.001) but not ghrelin (*p* = 0.249) or PYY (*p* = 0.361). No interaction effects were observed for ghrelin (*p* = 0.09), GLP-1 (*p* = 0.387), insulin (*p* = 0.422), PP (*p* = 0.153) or PYY (*p* = 0.422). Gut derived hormone data is presented in Figure 5.

## 4. Discussion

The aim of this investigation was to assess the effect of exercise intensity on subjective appetite, gastric emptying, blood metabolites and appetite-regulating hormones in healthy un-trained males. The main findings were that exercise intensity had little effect on gastric emptying rate, appetite or gastrointestinal hormone response despite some minor deviations at some time points. This suggests that exercise intensity is unlikely to have a significant effect on energy intake in the period after exercise despite the increased energy expenditure that occurs at higher exercise intensities.

To our knowledge, this is the first study to examine the gastric emptying rate of a standardised semi-solid meal and the responses of numerous appetite-regulating hormones after continuous aerobic exercise at different intensities. Previous studies have reported exercise intensity >60% $\dot{V}$O_2max_ results in suppression of appetite in untrained individuals [5,13,14,15]. In the present study, some subjective measures of appetite were marginally suppressed immediately post-exercise in the HIGH and LOW trials, yet no significant difference was observed at subsequent time points post-exercise nor post-meal. A limitation of the present study is the short 2 h monitoring period after exercise, as a longer monitoring period after exercise may offer more information on longer term appetite regulation. Food intake was not measured post-trial or over the following 24 h, so potential compensation in energy intake cannot be estimated. Rocha et al. [27] demonstrated compensation in energy intake immediately post trial and three days after an acute bout of aerobic exercise. Further investigation is required to clarify subsequent energy intake and whether this response differs if the energy expenditure for exercise is matched.

Appetite suppression immediately post-exercise has been observed in non-athletic populations [5,13], but this suppression has not always been reported. Holliday et al. [28] reported that, in endurance-trained males ($\dot{V}$O_2max_ = 61.6 ± 6.0 mL/kg/min), no significant reductions in subjective appetite were seen when participants completed a bout of a high intensity aerobic exercise. Consistent with these findings, King et al. [10] recruited trained males $\dot{V}$O_2max_ (55.9 ± 1.8 mL/kg/min) and found no difference in appetite between a control trial and brisk walking. The present study supports these observations as healthy un-trained males did not show differences in appetite when a standardised semi-solid meal is consumed instead of an *ad libitum* meal post exercise.

Gastric emptying rate in humans has been shown to be affected by ingested volume, nutrient content and may be influenced by previous dietary intake [29,30,31]. This is the first study, to the best of the authors’ knowledge, to have investigated gastric emptying responses to a semi-solid meal after continuous aerobic exercise at different intensities. Evans et al. [23] observed that exercise intensity appeared to have little effect on gastric emptying rate of a glucose solution after the completion of the exercise stimulus. The results of this study support these observations. Gastric emptying rate is regulated by splanchnic blood flow and central nervous system activity during exercise while some circulating gut-derived hormones have been shown to influence this variable. It would seem as though the changes in splanchnic blood flow and central nervous system activity that occur during exercise are likely to return to pre-exercise levels relatively quickly after completion of exercise, thus having less of an influence on gastrointestinal function in recovery. In this study, little difference was observed in circulating gut-derived hormone response after exercise, which could also explain the lack of difference in gastric emptying rate of the test meal. Some notable limitations must be acknowledged. The meal challenge provided was in accordance with a single serving suggested by the food manufacturers and, as such, the energy content provided was relatively low. If this were to be increased, some subtle difference in gastric emptying rate may be observed. This is in agreement with the results of Moore et al. [32], who reported that the volume and energy density of the meal is crucial to the speed at which gastric emptying occurs. The rate is exponentially related to the volume in the stomach; therefore, the fuller the stomach, the faster the rate of emptying. Secondly, *ad libitum* water consumption during the trial was significantly different with the largest fluid consumption within the HIGH and the lowest in the CONTROL, as would be expected. This difference in fluid intake may have effected gastric emptying rates; however, whether these fluid results in the present study indicate a potential mechanism for the results observed is questionable as the influence of hydration level during exercise on gastric emptying rate is unclear. This suggests exercise intensity, at least between 40–70% $\dot{V}$O_2Peak_, has no effect on gastric emptying rate of a semi-solid meal.

No significant differences between exercise intensities for blood glucose, NEFA, total cholesterol and triglycerides concentrations were observed in the present study. These findings are consistent with previous research that also reports significantly higher glucose concentrations 30–60 min after food ingestion [10,33]. Interestingly, Clayton et al. [33] investigated 24 h severe energy restriction (providing on 25% of energy requirements) and revealed that following 24 h energy restriction, similar to the CONTROL trial in the present study, plasma glucose concentration 60 min after consuming a standardised breakfast was increased compared to an energy balanced control trial. Within the current study, NEFA increased from pre to post exercise in both exercise conditions. Aligned with previous literature [13,33,34], NEFA concentrations fall after the ingestion of food containing carbohydrate, due to its stimulatory effect on insulin release. Triglyceride concentration was unchanged, with no differences between trials regardless of exercise intensity. Triglycerides usually increase within the blood in response to changes in energy demands [35]. The lack of difference between the trials is to be expected as gastric emptying rate was unaffected by exercise intensity and thus nutrients would have been available for absorption at a similar rate.

No significant differences were observed for gut peptide concentrations (ghrelin, GLP-1, PYY and PP) or insulin). Although ghrelin acts as an appetite stimulant, within the present study, acylated ghrelin responded to exercise intensity differently, as serum concentrations decreased by ~27% in the HIGH and increased by ~12% in the LOW. This decrease in ghrelin has consistently been observed within the literature for exercise intensity >70% $\dot{V}$O_2max_ [4,8,20], although these changes were not significantly different post exercise in this study. A limitation must be acknowledged, as sample numbers within the hormone measurements were relatively low (*n =* 8) due to complications with blood sampling. This is a common issue within research of this nature [4], and future research within this area must consider larger sample size. However, despite these changes showing no significance, previous research has also failed to observe an immediate difference in ghrelin following an acute bout of exercise [5]. Investigations have shown an inverse relationship between ghrelin and insulin, with postprandial rise in insulin [36,37,38,39]. This was not observed within the current study, as no changes in ghrelin were seen regardless of the rise in insulin concentrations post-meal. The findings for GLP-1 and PP are consistent with previous literature regarding elevated levels following food consumption [40,41,42]. However, within the current study, the increase 30 min post meal was not significant. There was also a lack of variation for PYY within the current study. However, De Silva and Bloom [43] suggest that PYY may be increased dependent on the energy content of the meal consumed. Therefore, the PYY findings within the current study may be due to the relatively small standardised semi-solid lunch provided. These findings suggest that increasing the exercise intensity potentially represents a viable strategy to increase the likely negative energy balance augmented by exercise, without increasing short-term appetite later in the day. If subsequent metabolic responses are sustained in the long-term, this could have important implications for weight management.

## 5. Conclusions

In conclusion, the post-exercise gastric emptying rate was similar regardless of exercise intensity. There was no effect of exercise intensity on subjective appetite after consuming a standardised semi-solid meal post-exercise. While some minor deviations in gut hormone response were noted, these were not statistically different between trials. These findings suggest that exercise intensity does not affect gastrointestinal measurements within a 2 h recovery period. Furthermore, the additional significant physiological demands of the high intensity trial did not result in compensatory alterations in the hormonal regulation of appetite or subjective appetite responses in the time period measured. Future research is required to investigate the mechanisms responsible for changes in appetite, gastric emptying and circulating hormones as a consequence of different forms of exercise (continuous, intermittent or energy expenditure matched).
